# The mouse motor system contains multiple premotor areas and partially follows human organizational principles

**DOI:** 10.1016/j.celrep.2024.114191

**Published:** 2024-05-07

**Authors:** Alberto Lazari, Mohamed Tachrount, Juan Miguel Valverde, Daniel Papp, Antoine Beauchamp, Paul McCarthy, Jacob Ellegood, Joanes Grandjean, Heidi Johansen-Berg, Valerio Zerbi, Jason P. Lerch, Rogier B. Mars

**Affiliations:** 1 Wellcome Centre for Integrative Neuroimaging, FMRIB, Nuffield Department of Clinical Neurosciences, University of Oxford, Oxford, UK; 2 DTU Compute, Technical University of Denmark, Kongens Lyngby, Denmark; 3 A.I. Virtanen Institute for Molecular Sciences, University of Eastern Finland, 70150 Kuopio, Finland; 4 NeuroPoly Lab, Institute of Biomedical Engineering, Polytechnique Montreal, Montreal, QC, Canada; 5 Mouse Imaging Centre, The Hospital for Sick Children, Department of Medical Biophysics, University of Toronto, Toronto, ON, Canada; 6 Bloorview Research Institute, Holland Bloorview Kids Rehabilitation Hospital, Toronto, ON, Canada; 7 Donders Institute for Brain, Cognition and Behaviour, Radboud University Nijmegen, Nijmegen, Netherlands; 8 Neuro-X Institute, School of Engineering (STI), EPFL, 1015 Lausanne, Switzerland; 9 CIBM Center for Biomedical Imaging, 1015 Lausanne, Switzerland

## Abstract

While humans are known to have several premotor cortical areas, secondary motor cortex (M2) is often considered to be the only higher-order motor area of the mouse brain and is thought to combine properties of various human premotor cortices. Here, we show that axonal tracer, functional connectivity, myelin mapping, gene expression, and optogenetics data contradict this notion. Our analyses reveal three premotor areas in the mouse, anterior-lateral motor cortex (ALM), anterior-lateral M2 (aM2), and posterior-medial M2 (pM2), with distinct structural, functional, and behavioral properties. By using the same techniques across mice and humans, we show that ALM has strikingly similar functional and microstructural properties to human anterior ventral premotor areas and that aM2 and pM2 amalgamate properties of human pre-SMA and cingulate cortex. These results provide evidence for the existence of multiple premotor areas in the mouse and chart a comparative map between the motor systems of humans and mice.

## Graphical abstract

**Graphical abstract:**
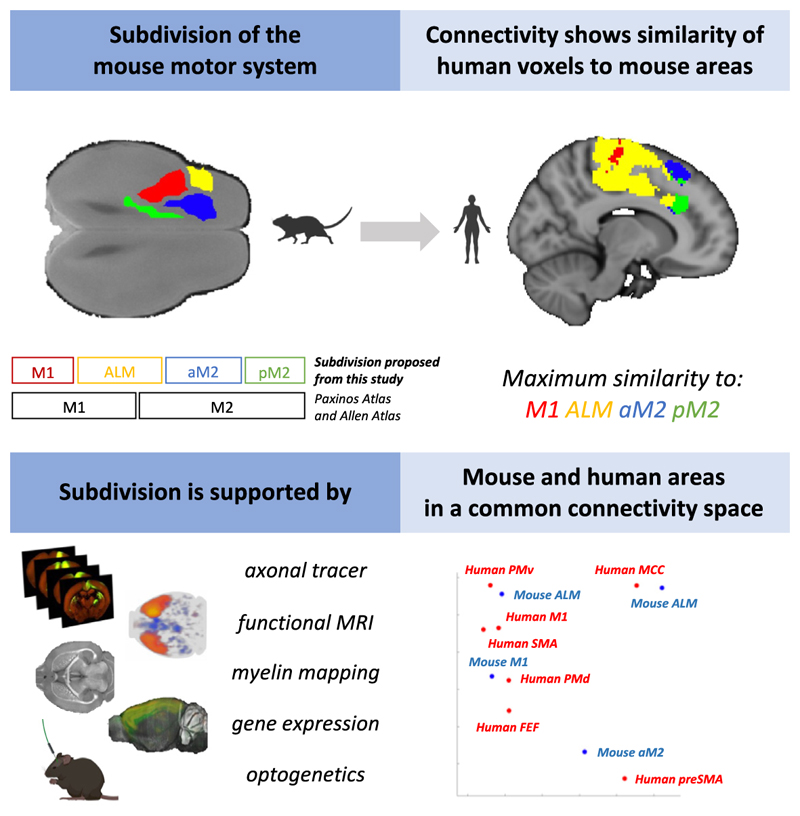

## Introduction

Premotor circuits are fundamental for motor behaviors in health and disease. In humans, premotor areas are known to be actively involved in both movement execution and motor learning.^1^ It has also long been established that plasticity of premotor circuits is crucial in supporting motor rehabilitation in patients with stroke.^2–7^ Indeed, premotor circuits are also common targets for intervention.^8,9^ As such, it would be beneficial for the discovery and validation of novel treatments to be able to study these circuits in mouse models, which allow much more direct neuronal, pharmacological, and genetic manipulation of the system. However, similarity in organization of the motor systems between the human and the rodent brain is far from established,^10^ with several cortical areas in humans being considered premotor (such as ventral premotor cortex [PMv] and supplementary motor area [SMA]), but their similarity to rodent cortical areas is still a matter of debate.^11,12^

Premotor circuits in rodents have long been the subject of intense debate, with controversy surrounding (1) their location and subdivisions and (2) their similarity to human premotor areas. In particular, the exact location of the border between the primary motor area (MOp) and secondary motor area (MOs or M2) is controversial,^11,13–15^ as is the existence of subdivisions within MOp^16–22^ and MOs.^23–25^ Moreover, homology between mouse MOp and human primary motor cortex (M1),^10^ and between mouse MOs and various human premotor areas,^11,12,20,23,26,27^ remains mostly speculative.

Many of these controversies could not be readily settled until recently, as studies would rely on comparisons of the two species’ brains using data obtained through different means. For instance, invasive tracer studies are used to finely depict connections in the mouse brain,^28^ but these techniques are not available in humans. Recently, however, this problem has started to be addressed using neuroimaging.^29^ Magnetic resonance imaging (MRI) techniques sensitive to multiple aspects of brain organization, including the presence of large white matter bundles,^30^ functional connectivity measured using resting-state functional MRI (rs-fMRI),^31,32^ and tissue properties such as cortical myelin measured using quantitative MRI (qMRI),^33^ can be employed in an identical manner in multiple species, allowing a comparison of like with like across species. These techniques have been particularly successful in comparing human and non-human primate brains, leading to a number of insightful human-macaque comparisons,^30,34^ and contributing to a growing literature on mapping brain regions across species.^12,35–37^

**Figure 1:**
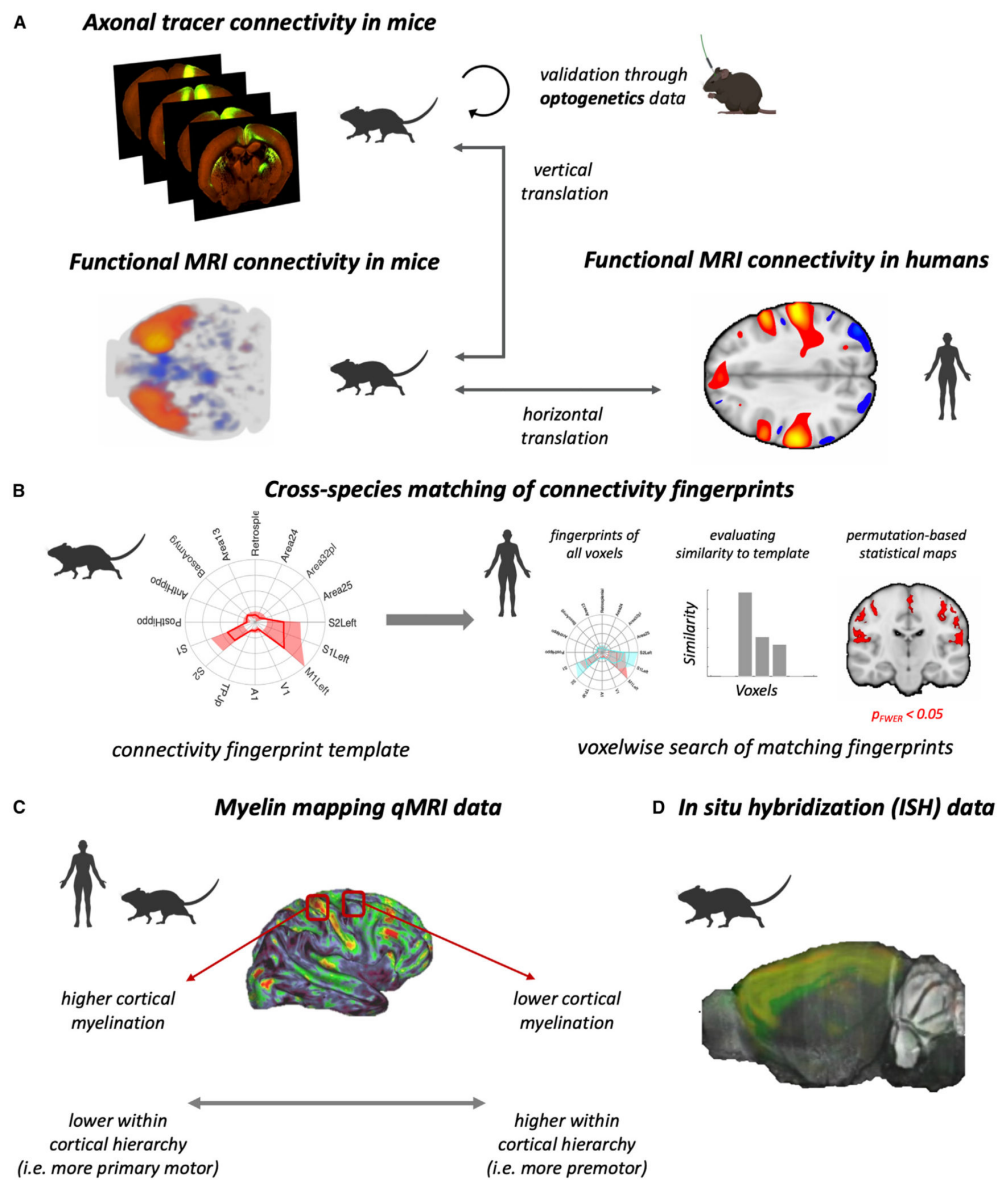
Multimodal data to study the motor system across species (A) Whole-brain, fine-grained axonal connectivity data, available in mice but not in humans, can be used to study the neuroanatomy of motor and premotor areas in the mouse. rs-fMRI connectivity data are available in both mice and humans, thus allowing for a comparison of axonal and fMRI results in the mouse (vertical translation) and then comparing the connectivity of mice and humans (horizontal translation). (B) The fingerprint matching technique enables reliable comparison of rs-fMRI connectivity across species. (C) Myelin mapping qMRI data allow us to compare cortical hierarchy of the motor system across species.^42–44^ (D) Genetics data allow us to compare gene expression profiles of the motor systems across species.

More recently, advances in rodent functional imaging^38–40^ have opened up the possibility of similar direct comparisons between the rodent and the human brain.^41^ Here, we aimed to generate a quantitative, data-driven understanding of mouse motor and premotor areas, i.e., the mouse motor system, and of its relationship with the human motor system. To this end, we used “ground-truth” axonal tracer data to perform a parcellation of the extended territory of the motor system in the mouse. Having established the anatomical subdivisions of the mouse motor system, we then used neuroimaging to establish the similarities and differences of motor system organization between mice and humans (Figure 1).

## Results

In this study, we used axonal tracer data from the Allen Mouse Brain Connectivity Atlas^28^ to perform a parcellation of the mouse motor system (Figure 1A). To further validate the parcellation, and to compare properties of motor areas across species, we relied on several complementary datasets: a spatial library of optogenetics experiments in the mouse (Figure 1A, as per Le Merre et al.^24^); rs-fMRI connectivity data (Figures 1A and 1B) in the human (from the Human Connectome Project)^45^ and the mouse (as per Zerbi et al.^46^); myelin mapping qMRI data (Figure 1C) in the human (as per Lazari et al.^9^) and the mouse (newly collected for this study); and gene expression data in the mouse (Figure 1D) from the Allen Mouse Brain Atlas.^47^

### Clustering of axonal tracer data reveals multiple anatomically and functionally distinct subdivisions within the mouse motor system

**Figure 2:**
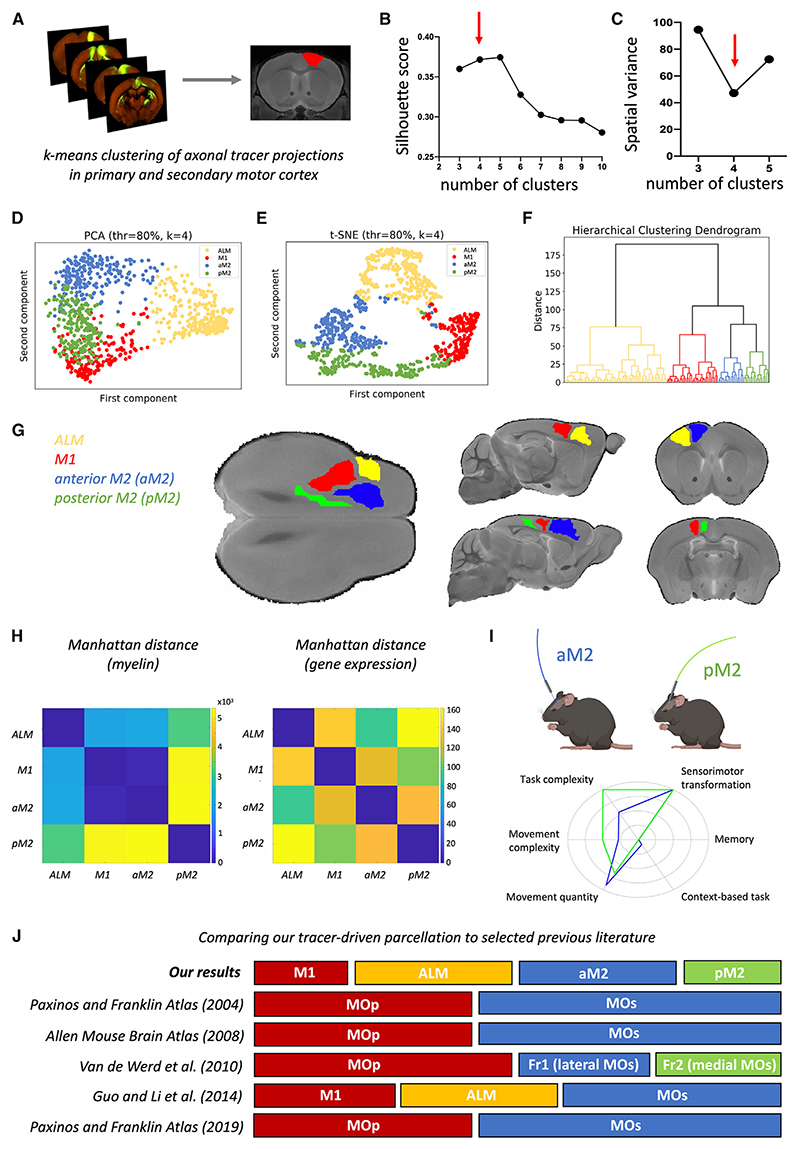
Data-driven parcellation of the mouse motor system based on axonal tracer data (A) Axonal tracer data are used to derive motor and premotor subdivisions in a data-driven way through k-means clustering. (B) Silhouette score for different numbers of clusters/subdivisions. (C) Subdivisions are most spatially homogeneous for a 4-cluster subdivision. (D) Principal-component analysis (PCA) plot of the 4-cluster subdivision. (E) t-Distributed stochastic neighbor embedding (t-SNE) plot of the 4-cluster subdivision. (F) Hierarchical clustering plot of the 4-cluster subdivision. (G) 3D location of the k-means-derived subdivisions. (H) Cortical myelin and gene expression differences between the 4 k-means-derived subdivisions. (I) Based on 24 previous optogenetics studies (15 targeted aM2 and 9 targeted pM2), we summarize the involvement of aM2 and pM2 in different types of behavior. The axes represent the normalized percentage of studies showing an effect of optogenetic stimulation. (J) Schematic diagram comparing our data-driven subdivision of the motor system to that of previous literature

As the exact location and subdivision of MOp and MOs/M2 in the mouse are still controversial, we first used axonal-tracer-based connectivity data to derive connectivity-based parcels of the motor system using k-means clustering (Figure 2A) and find an optimal parcellation made of 4 areas: anterior-lateral motor cortex (ALM; corresponding mostly to the anterior part of traditional MOp, with 74.72% of the voxels falling within MOp and 25.28% of voxels falling within MOs), M1 (corresponding to the posterior part of traditional MOp), anterior M2 (aM2; corresponding to the anterior-lateral part of traditional MOs), and posterior M2 (pM2; corresponding to the posterior-medial part of traditional MOs). Similarities to and differences from selected previous literature^15,17,25,48,49^ are further summarized in Figure 2L. The respective coordinates of the center of the four areas in Allen Mouse Brain Common Coordinates^49,50^ are roughly as follows: M1, −0.2 AP, 1.5 ML, 1.5 DV; ALM, 1.7 AP, 2 ML, 1.5 DV; aM2, 1.5 AP, 0.6 ML, 1.5 DV; and pM2, −0.2 AP, 0.4 ML, 1.5 DV. To facilitate identification of the areas within commonly used anatomical spaces, we have also indicated their putative locations (Figure S1) within the Paxinos stereotaxic space^15^ and made this updated atlas openly available.

In particular, we found that solutions describing three or more clusters within the motor network provide the most stable and reliable spatial geometry (Figures 2B–2F; Video S1). Of the stable parcellations, three parcellations have the highest silhouette score (Figure 2B), with 3 (two MOp parcels, one MOs parcel), 4 (two MOp parcels, two MOs parcels), and 5 clusters (three MOp parcels, two MOs parcels). Among these 3 parcellations, the k = 4 solution lies at the elbow of the inertia plot (Figure S2) and has the lowest variance in the size of its parcels (Figure 2C). We found that these results are remarkably consistent across different data preprocessing steps (Figure S2) and that they match previous literature outlining the presence of two subclusters for both MOp and MOs.^17,20,23,25^ Therefore, we adopted the k = 4 parcellation for all further analyses.

We found a border running in the anterior-posterior direction between MOp and MOs, with MOp being fully lateral to MOs (Figure 2G). This border is coherent with recent literature^14^ and has a high degree of overlap with the one in existing atlases such as the DSURQE atlas,^51^ the Paxinos atlas,^15^ and the Allen Atlas,^50^ which provides further confirmation of current divisions between MOp and MOs.

Within MOp, we found a separation between anterior and posterior subdivisions, in line with previous literature^17–19^ reporting a difference between the ALM area and the M1 proper (this subdivision has also been reported as the rostral forelimb area [RFA] and the caudal forelimb area [CFA]^20–22^) within the MOp. As such, we will refer to these areas as ALM and M1.

Within MOs, we found a border separating the anterior-lateral component of MOs from the posterior-medial component; as such, we will refer to these areas as aM2 and pM2. It is note-worthy that the location of the border within MOs provides an explanation for mismatch in previous literature. While previous papers investigating individual 2D slices found evidence for either an anterior-posterior^23^ or a medio-lateral^25^ border within MOs, this subdivision suggests that both views are true, depending on how the tissue is sliced.

We next sought to corroborate these results through independent datasets that explored non-connectivity features of mouse cortical areas, namely, gene expression profiles and cortical myelin content. To this end, we examine openly available gene expression profiles from 3,859 genes using permutation testing and find significant differences in levels of gene expression between these putative subdivisions (Figure 2H, ALM vs. M1: *p* < 0.001; ALM vs. aM2: *p* < 0.001; ALM vs. pM2: *p* < 0.001; M1 vs. aM2: *p* < 0.001; M1 vs. pM2: *p* < 0.001; aM2 vs. pM2: *p* < 0.001). We then adapted a multi-parameter mapping protocol^52^ for 7T MRI scanning of mouse brains (Figure S3) in order to collect myelin markers.^53–57^ We find that myelin markers in pM2 are significantly different from M1, ALM, and aM2 (Figure 2H, all *p* < 0.001), whereas other areas do not differ significantly from each other (ALM vs. M1: *p* = 0.7059; ALM vs. aM2: *p* = 0.5577; M1 vs. aM2: *p* = 0.3499). Taken together, these results indicate that the anatomical subdivisions we uncovered differ not just in their connectivity to other areas but also in their gene expression levels and, to a lesser degree, in their intrinsic cortical myelin content.

Anatomical subdivisions are also likely to have distinct functional profiles, which can be tested by investigating the results of local inactivations on behavior. Indeed, inactivations of M1 and ALM are already well established to produce distinct behavioral deficits,^20^ with ALM producing deficits in higher-order motor planning.^18,19,23^ This was further confirmed by a recent optogenetic screen across MOp, which focused on licking behavior.^58^ However, differential outcomes from inactivations of aM2 vs. pM2 have not been previously tested.

To test whether inactivation of aM2 vs. pM2 produces different behavioral outcomes, we surveyed an existing openly available database of 100 previously published inactivation experiments targeting mouse frontal areas and establishing impairments in a wide range of task-based behaviors.^24^ We found 24 studies in total that targeted coordinates within MOs (full description in Table S1). Based on their reported stereotactical coordinates, we were then able to determine that 15 targeted aM2 (with a pooled sample size of 120 mice) and 9 targeted pM2 (with a pooled sample size of 84 mice). Using prespecified task descriptors from the original database,^24^ we then explored which task features were associated with impairments by aM2 and pM2 inactivation. We found that both aM2 and pM2 inactivations are reported to interfere with sensorimotor transformations (Figure 2I). pM2 has been reported as more frequently interfering with complex tasks and tasks involving complex movement, whereas aM2 affects more frequently tasks that are context dependent and involve multiple movements (Figure 2I). While the findings are based on individual perturbation studies of aM2 and pM2 and should be further validated with side-by-side comparison experiments, this analysis of data from 208 mice provides an initial suggestion of a behavioral specialization for aM2 and pM2 and generates new hypotheses on their respective roles in complex behaviors. Taken together, our results indicate a marked difference between subregions of MOs across axonal connectivity, microstructural, and behavioral assays.

### Functional connectivity differences between mouse premotor areas match predictions from axonal tracer data

While axonal tracer connectivity can provide detailed ground-truth measurements for connectivity in the mouse, such measurements are not available in humans. Therefore, we sought to further validate the findings from axonal tracer data with rs-fMRI, which is available in both mice and humans and would thus afford us with the ability to compare premotor connectivity across species.

**Figure 3:**
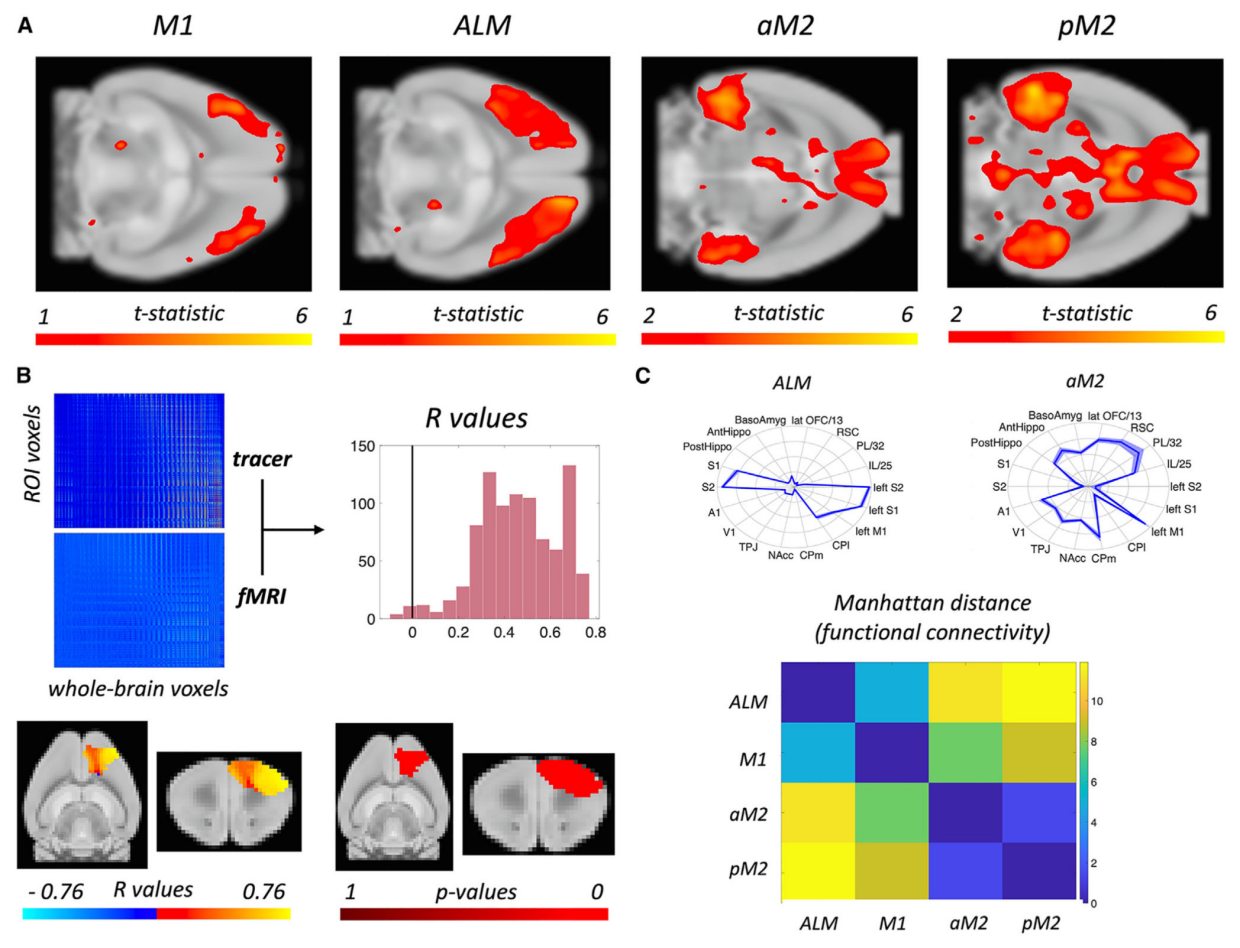
Bridging axonal and rs-fMRI connectivity in the motor system of the mouse (A) Four subdivisions of the premotor-motor network in the mouse have different patterns of rs-fMRI functional connectivity. (B) Broad agreement between whole-brain tracer and rs-fMRI connectivity within the mouse premotor-motor network. (C) After functional connectivity patterns are reduced to connectivity fingerprints, differences between subdivisions of the premotor-motor network are still present.

We first examined the rs-fMRI whole-brain connectivity profiles of M1, ALM, aM2, and pM2. We observed different patterns of connectivity across the whole brain (Figure 3A), indicating that differences between subdivisions of the motor network persist in rs-fMRI connectivity data. In order to test for similarities between axonal tracer and rs-fMRI connectivity in the mouse, we then tested how the connectivity between each subdivision and the voxels in the rest of the brain correlate across these two modalities. More specifically, for each voxel within the mouse motor network, we calculated the Pearson correlation coefficient and related *p* value between its tracer-based connectivity and its fMRI-based connectivity values. We find that over 99% of voxels in the premotor-motor network show significant correlations (all below *p* < 0.001, rejecting the null hypothesis that there is no correlation between fMRI connectivity and tracer connectivity in that voxel), with a mean effect size of R = 0.4587 (Figure 3B). To build a conservative null distribution, we also calculated correlation coefficients between MOp and MOs fMRI connectivity profiles and tracer connectivity profiles from voxels picked at random outside of Mop and MOs. This null distribution has a mean R of −0.0126 and a median R of −0.0166, as opposed to the real distribution, which has a mean R of 0.4587 and a median R of 0.4598 (two-sample Kolmogorov-Smirnov test, *p* < 0.001). When looking at the spatial pattern of R values and *p* values (Figure 3B), we find them to be mostly uniform, with some variation in R values, which are highest in the anterior-lateral area and in the medial areas of the motor network. Taken together, these analyses show remarkable similarities between connectivity metrics within the motor and premotor circuits of interest.

As a further intermediate step toward comparison between mouse and human rs-fMRI data, we tested whether the rs-fMRI connectivity profile of each area within the mouse motor system could be expressed in terms of a “connectivity fingerprint” by calculating the connectivity of the area with a prespecified set of target areas across the brain whose homologies between mouse and human brain are known (Table S2; see STAR Methods for details).

These connectivity fingerprints (Figure 3C) showed substantial differences in connectivity across the mouse motor system. In particular, M1 and ALM are strongly connected to sensorimotor areas (such as S1, S2, and contralateral M1) but show lower connectivity to hippocampus, amygdala, and higher-order areas (such as retrosplenial, infralimbic, and orbitofrontal cortex). aM2 and pM2, by contrast, show an opposite pattern, with strong connections across hippocampus, amygdala, and higher-order areas but little connection to somatosensory cortices. M1 and ALM are further separated by their relative level of sensory and motor connectivity, with M1 being more strongly connected to contralateral M1. aM2 and pM2 are further separated by their relative levels of restrosplenial connectivity. Similarly to whole-brain results, we find significant differences between the connectivity profiles of the four subdivisions of interest (ALM vs. M1: *p* < 0.001; ALM vs. aM2: *p* < 0.001; ALM vs. pM2: p = 0.0016; M1 vs. aM2: *p* = 0.0014; M1 vs. pM2: *p* < 0.001; aM2 vs. pM2: *p* < 0.001).

### Comparison of the motor system across mouse and human brains

Having validated rs-fMRI connectivity measures in our areas of interest through comparison of axonal tracer data and rs-fMRI functional connectivity data (“vertical translation,” see Mars et al.^29^), we next sought to use rs-fMRI data across species to identify similarities and differences between human and mouse premotor circuits (“horizontal translation”). Our approach relies on the fact that each motor system area is described in terms of its connectivity with areas whose homologs are known across the mouse and human brains. In other words, the homologous areas form a common connectivity space in which both the mouse and human (pre)motor areas can be described and quantitatively compared.^29,59^

**Figure 4:**
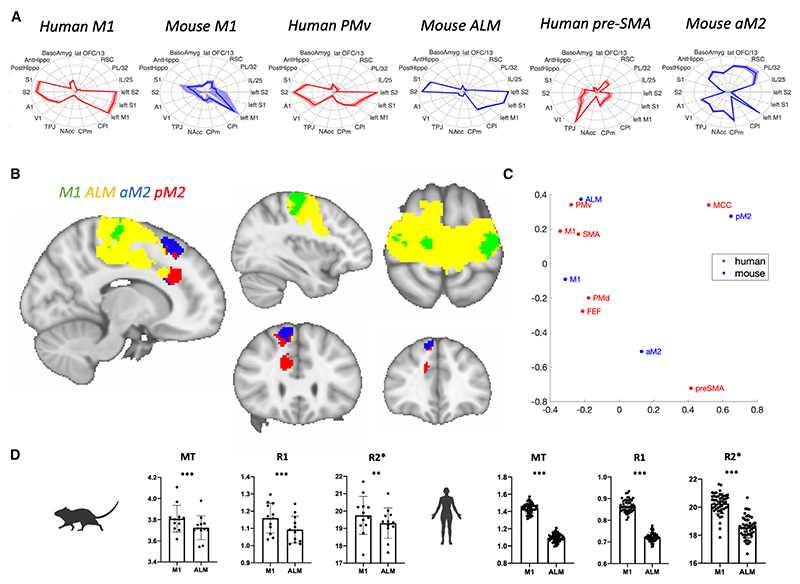
Connectivity-based comparison of human and mouse motor systems (A) Connectivity fingerprints of mouse and human areas. (B) Winner-takes-all map of mouse-to-human similarity. Each voxel’s color represents the mouse cortical area to which the voxel’s connectivity is most similar. (C) Spectral clustering. A graphical representation of the distance between cortical areas of mice and humans in connectivity space is shown. (D) Mapping of cortical myelin across species. Mouse M1 has higher values on all three makers than ALM (MT: t = 5.453, *p* = 0.0002; R1: t = 7.989, *p* < 0.0001; R2*: t = 3.931, *p* = 0.0024; *n* = 12, mean ± SD, paired sample t test). Similarly, human voxels similar to mouse M1 have higher values than voxels similar to ALM (MT: t = 66.07, *p* < 0.0001; R1: t = 55.14, *p* < 0.0001, R2*: t = 21.46, *p* < 0.0001; *n* = 50, mean ± SD, paired sample t test). ***p < 0.001 and **p < 0.01.

Similar to the mouse motor system, human motor system areas also have substantial differences in connectivity, as evidenced by their connectivity fingerprints (Figure 4A). Posterior areas such as M1 and PMv have high connectivity to sensory areas (such as S1, S2, and V1) but low connectivity to hippocampus, amygdala, and higher-level areas (such as retrosplenial and prefrontal cortex). Posterior areas are further separated by their relative level of sensory and motor connectivity, with M1 being more strongly connected to contralateral M1 than to sensory areas. Anterior areas, such as pre-SMA and motor cingulate cortex, have strong connections to temporo-parietal areas and to retrosplenial cortex and weaker connections to contralateral motor cortex and to sensory areas.

As a first step, we compared the connectivity profiles of the four mouse motor system areas to the connectivity profile of each voxel in a large region of interest consisting of human areas M1, FEF, PMd, PMv, SMA, pre-SMA, and cingulate motor areas (RCZa and RCZp). This region of interest is based on previous literature^31,60^ and spans the precentral gyrus as well as clustering-based parcellations of pre-SMA, SMA, and mid-cingulate cortex. We then combined information across these four individual analyses (Figure S4) through a winner-takes-all algorithm to assign each human premotor and motor voxel to its most similar mouse counterpart.

The resulting map (Figure 4B) shows that mouse M1 has the closest human correspondence with the hand-knob M1 area of the precentral gyrus. Importantly, compared to voxels belonging to ALM, voxels belonging to mouse M1 clustered together spatially in human M1 proper, at the posterior end of the gyrus, while voxels most similar to ALM located in the anterior and inferior aspects of the gyrus, including large parts of ventral area 6 overlapping with the territory of PMv. Although both M1 and ALM are subdivisions of the mouse M1 as defined by most current neuroanatomical atlases,^15,51^ the stronger similarity of ALM lies within human PMv.

Apart from connectivity, human motor and premotor regions can be distinguished based on their tissue properties, some of which can be detected using myelin markers from qMRI.^43,54,55^ More broadly, myelin markers have been previously used to study neuroanatomical subdivisions across species^42,61,62^ and within species, including in human^43^ and in mouse.^44^ We therefore used three myelin-sensitive markers—MT, R1, and R2*—and compared them across mouse M1 and ALM and across human voxels with a connectivity profile most similar to mouse M1 and ALM (Figure 4D). This showed that mouse M1 had higher values on all three makers than ALM (MT: t = 5.453, *p* = 0.0002; R1: t = 7.989, *p* < 0.0001; R2*: t = 3.931, *p* = 0.0024). Importantly, this result was mimicked in the human, with mouse-M1-like voxels showing higher values than ALM-like voxels (MT: t = 66.07, *p* < 0.0001; R1: t = 55.14, *p* < 0.0001, R2*: t = 21.46, *p* < 0.0001). These results confirm that, for this part of the motor system, similarity in cortical organization between mice and humans is present not only in connectivity but also in tissue properties.

Returning to the connectivity comparison (Figure 4B), aM2 shows similarity to the superior subdivision of pre-SMA, whereas pM2 shows similarity to the anterior subdivision of pre-SMA and to MCC. M2 has previously been proposed to be a homolog of PMv,^11^ SMA,^26,27^ or M1^63,64^ or an amalgam of human premotor areas.^12^ Our result best match the amalgam theory, with aM2 and pM2 not being straightforward analogs of pre-SMA but rather integrating properties of MCC as well. It is worth noting that while mouse M1 and ALM have large areas of significantly similar voxels to human, aM2 and pM2 do not (Figure S5), which further points to a stronger similarity between human and premotor circuits within human M1 and PMv than in human dorsomedial premotor areas such as pre-SMA.

To further visualize the similarities between human and mouse motor systems, we use a spectral embedding algorithm^65^ to display all human and mouse areas together in a 2D connectivity space (Figure 4C). We find that human M1, PMv, and SMA cluster together, with the more dorsolateral premotor regions PMd and FEF forming a separate cluster. MCC is separate, and the most prefrontal region of the human motor network, pre-SMA, forms another completely separated extreme. Interestingly, mouse M1 and ALM show the greatest proximity to traditional human motor and premotor regions, while mouse pM2 and aM2 are more closely associated with the extremes of MCC and pre-SMA, respectively. As a further sensitivity analysis, given that visuomotor connections have been postulated to be different in primates due to primate-specific visually guided grasping behavior that is absent in other species,^66–68^ we repeated the spectral clustering analysis on connectivity fingerprints, leaving out information from both V1 and A1, and found similar results (Figure S5).

As a control analysis, we selected the inferior temporal gyrus as a region of interest, given its lack of direct spatial and functional relation to the motor network, and tested for similarity to various mouse fingerprints (Figure S6). We found that no voxels in the human inferior temporal gyrus had a connectivity similar to that of mouse M1, aM2, and pM2; only a handful of voxels showed some similarity to ALM (4.9% of all voxels tested), albeit with lower effect sizes compared to previous analyses (peak_t-stat_ = 4.85), thus reinforcing the specificity of the main effects reported. Finally, we also confirmed that our results are similar when using a larger sample size of 100 subjects (Figure S7).

In summary, while M1 and ALM are most similar to classic human motor and premotor regions, mouse M2 subdivisions occupy a position closer to human medial frontal regions, with aM2 in particular occupying a position close to a human prefrontal region.

## Discussion

In the past two decades, many promising pharmacological treatments for patients with stroke, first identified in rodent studies, have failed to deliver benefits in human clinical trials.^69,70^ While the reasons for this are varied and may go beyond neuroanatomical differences across species,^69,71,72^ a crucial obstacle for the translation of preclinical findings is the lack of established similarities—and differences—between the human and rodent motor systems. Premotor cortex, for instance, is known to be important in human stroke recovery,^3,5^ but the location and existence of a rodent homolog for premotor cortex has been long debated. Here, we attempt to further the field by (1) providing a more detailed characterization of the mouse premotor system and (2) assessing how the organizational principles of the human and mouse premotor systems compare. Our findings show that both MOs and MOp can be further subdivided into regions that have distinct connectivity, gene expression, and cortical myelin content and whose inactivation leads to differential behavioral results. Some organizational principles of human and mouse premotor systems are comparable, but there were also noticeable differences. We will discuss our results and their implications and limitations in detail below.

Our findings highlight that the mouse motor system is more heterogeneous than previously appreciated. While MOp is sometimes described as a unitary cortical area,^15,50,73^ our results are aligned with substantial evidence for a subdivision between anterior and posterior areas of MOp, often described as RFA and CFA.^20–22^ In particular, there has been an influential body of work investigating the circuitry of RFA and its role in movement preparation but renaming it ALM area due to a small overlap with M2.^17–19^ Our findings provide further evidence for the existence of a premotor area within MOp, which we have referred to as ALM to be consistent with the most recent literature.

Our study also finds evidence for a much-debated subdivision within MOs. One early report of a boundary between lateral and medial M2^25^ had been contradicted by several strands of evidence.^24,26,74^ However, recent widefield calcium imaging and optogenetics studies have highlighted a potential difference between anterior and posterior MOs across a range of tasks.^16,23,75,76^ Our results reconcile these findings by showing the presence of an antero-medial border within MOs. The fact that this border runs at an angle to the midline may help explain the inconsistency of previous results: depending on which slice is being considered in coronal histological slices, or which cortical patch is being considered in electro-physiology studies, MOs may have wrongly appeared homogeneous or to have a solely posterior or solely lateral border.

It is also noteworthy that the exact locations of MOs and MOp have also proven controversial,^13^ with different sources describing MOs as lateral to MOp,^11^ frontal to MOp,^73^ or posterior-lateral to MOp.^15^ Our results argue for the first hypothesis and find an anterior-posterior border between MOp and MOs, with MOs being fully lateral to MOp. This border has a high degree of overlap with recent literature^14^ and with most existing atlases, such the Paxinos atlas,^15^ the Allen Atlas,^50^ or the Ullman cortical parcellation, which is incorporated in the DSURQE atlas.^51^

Are the mouse and human premotor systems similar to each other? We show that for what concerns mouse M1 and ALM, there are numerous cross-species similarities to human M1 and human lateral premotor areas, including PMv. This result is somewhat surprising, as close mouse homology to human PMv has been previously ascribed to M2,^11^ and some studies have suggested that PMv may a primate invention with no mouse homolog.^66–68,77^ Moreover, while it is well established that ALM has a premotor function,^17,58,78–80^ premovement preparatory dynamics in the human brain are distributed across multiple areas,^81,82^ making it difficult to disentangle which human premotor areas have similar properties to ALM based purely on task-driven preparatory dynamics. Our results provide a data-driven argument for a similarity between human PMv and mouse ALM with respect to their connectivity profiles to homologous areas as well as their cortical myelin profiles. However, these results should not be taken as a claim of homology between the mouse and human areas but rather as a similarity in particular principles of their cortical organization.

Similar caution should be observed in the case of aM2 and pM2. MOs has been proposed to be a homolog of PMv/PMd^11^ or SMA^26,27^ or an amalgam of human premotor areas.^12^ Moreover, while human cingulate cortex also has a motor subdivision capable of directly eliciting movements via corticospinal connections,^83,84^ similarity between mouse M2 and human motor cingulate cortex had not been previously tested. Our results best match an updated version of the amalgam theory, with aM2 and pM2 not being straightforward homologs of medial frontal cortices, such as pre-SMA, but rather mixing connectivity properties of human premotor and motor cingulate cortex.

A key finding from our study is that no area of the mouse motor network has similar connectivity to human pre-SMA. Together with the fact that mouse aM2 and pM2 do not have a clear similarity to any single area in the human motor network, this raises new questions about the extent to which rodent models can be used in stroke research. In particular, it is known that frontal areas of the motor network, including pre-SMA, are involved in human stroke recovery.^2,7,85^ Moreover, M2 is currently the focus of many rodents studies focusing on motor learning^86,87^ and stroke.^88,89^ Therefore, it is possible that differences between human and mouse motor networks may have contributed to the low clinical translatability of promising treatments identified in rodent stroke models^69,70^ and may be an additional limiting factor in future preclinical studies focused on stroke recovery. While our study does not directly investigate stroke recovery, it suggests that the translatability of stroke studies could be improved by focusing on premotor areas of high similarity between humans and rodents, such as mouse ALM and M1 and human PMv and M1.

Overall, these results suggest that multimodal and quantitative data-driven analyses have great potential in resolving long-running neuroanatomical controversies about rodent neuroanatomy. In the human brain, fully data-driven parcellations have already helped resolve many debates on human neuroanatomy^90,91^ and have even allowed more precise targeting of brain stimulation in health^92^ and disease.^93^ Our study hints that a similar data-driven approach may prove fruitful in mice as well and that studying mouse neuroanatomy in greater detail could help resolve controversies surrounding other mouse areas such as cingulate cortex^94^ or prefrontal cortex.^95^

In conclusion, while M2 is traditionally considered to be the only higher-order motor area of the mouse brain and is thought to combine properties of various human premotor cortices, we have shown several lines of evidence contradicting this notion. In particular, we found that ALM, outside of M2, is most reminiscent of human PMv and that M2 has two further subdivisions. These results provide evidence for the existence of multiple premotor cortical areas in the mouse and reveal a greater degree of homology between the motor networks in humans and mice than previously described. Our study charts a path for forward translation (mouse to human) and reverse translation (human to mouse) of findings in the motor network. Neuroanatomically informed stroke studies in rodents may help improve forward translatability of findings to human stroke patient cohorts. Moreover, as the motor network is particularly well studied in humans,^96^ reverse translation to rodents holds the potential to open up many new avenues for mechanistic basic research bridging the two species.

### Limitations of the study

Our study combined two types of translational efforts. First, we assessed within the mouse whether our parcels, as determined by parcellation of tracer data, also showed differential profiles in other modalities. This is an example of what has been termed vertical translation.^29^ Then, we used the same modalities, based on neuroimaging data, to directly compare the rodent and human brain. This is an example of horizontal translation, which allows quantitative comparisons between species without the confounds of comparing different data types, which has proven troublesome in the past. Although horizontal translation has proven successful in comparisons between humans and non-human primates^30,34^ and between humans and mice,^41^ it does mean we are susceptible to some of the limitations associated with rs-fMRI data. First, we know that rs-fMRI is confounded by factors such as physiological noise. While we cannot fully exclude that spurious physiological correlations between areas may have driven some of our results, we have excluded this possibility to the best of our capacities by preprocessing the data with ICA-based denoising and validating our connectivity results in the mouse with anatomical tracer data. Second, rest is a behaviorally noisy, uncontrolled state, and rs-fMRI in rodents is further complicated by the use of anesthesia to improve functional connectivity estimates.^97,98^

Therefore, it is possible that analyzing connectivity during a motor task such as grasping may yield different results, in particular regarding areas that we currently could not find a rodent homolog for, such as human pre-SMA. However, task fMRI is not yet fully feasible in rodents due to technical limitations.^99^ Therefore, while additional task-based fMRI data may be available for mouse-human comparative studies in the future, rs-fMRI remains at present the best option for comparative studies of the rodent brain. As suggested by one reviewer, sample sizes can always be increased, and it would be interesting for future studies to look at samples sizes of over 10,000 subjects, which would allow additional analyses, such as comparisons across development,^100^ an issue that is of interest in the motor system.^101^

It should be noted that our division of the rodent motor system is based on a connectivity-based parcellation of invasive tracer data, the so-called “gold standard” of connectivity research. Connectivity-based parcellations of large parts of cortical territory have been a successful method for delineating separate areas.^31,91,102^ This approach is based on the logic that cortical areas can be distinguished by a unique set of connections,^103^ which often overlaps with cytoarchitecture^104^ and functional profile.^105^ Thus, although we cannot claim that our subregions correspond to distinct cytoarchitectonic areas at this time, the evidence provided indicates the presence of meaningfully distinct parcels. Moreover, our further analyses demonstrate that the subdivisions also differ in terms of resting-state functional connectivity, both whole brain and with a select fingerprint of human-mouse homologs; transcriptomic gene expression; cortical myelin content; and functional deficits following inactivation.

While we find evidence for extensive differences between subdivisions of the rodent motor system within the tissue and connectivity properties analyzed in this study, other properties may also vary across subdivisions. For example, optogenetic fMRI (opto-fMRI) may in the future allow probing the role of these subdivisions in whole-brain network physiology and brain activity dynamics,^106,107^ as well as the roles of distinct cell types within each subdivision in driving connectivity and functional differences.^106,108^ Moreover, our analyses on gene expression differences between motor network subdivisions were restricted to 3,958 genes available in the coronal dataset of the Allen Mouse Brain Atlas. While this dataset has been extensively used in neuroscience research^109–114^ and our selection and preprocessing of genes followed widely accepted guidelines,^115^ this collection of genes does not exhaustively cover all genes expressed in the mouse brain, which may have biased our results. Nonetheless, the genes selected are highly homologous to humans,^109^ which further supports their applicability for our translational analysis.

We have provided initial evidence of differences in behavioral deficits following inactivation of subdivisions of the mouse motor network. Nonetheless, optogenetics has a relatively limited spatial resolution of, at most, 1^116^ or 2 mm,^17,19,117^ and we cannot exclude that off-target effects from the stimulation of neighboring areas such as anterior cingulate cortex, ALM, or M1 took place during the studies we analyzed for aM2 and PM2. Therefore, our findings should be validated systematically with higher-resolution sub-millimeter techniques, such as multiphoton holographic optogenetics.^118^ Finally, the functional neuroanatomy of the motor network may also differ between male and female mice, which has not been explored in this study. Additionally, our study used data from mice and humans, but it is unclear whether the results would generalize to the motor network of other rodent species (such as rats) and other primates species (such as monkeys). Altogether, our data-driven subdivision of the murine motor network represents a foundation for further dissection of premotor and motor circuits across multiple species.

## Star⋆Methods

### Key Resources Table

**Table:** 

REAGENT or RESOURCE	SOURCE	IDENTIFIER
Deposited data		
Human resting-state fMRI	Human Connectome Project (HCP)	https://db.humanconnectome.org/
Mouse resting-state fMRI	(Zerbietal.)^119^	N/A
Human MPM	(Lazari et al.)^9^	N/A
Tracer Data	Allen Mouse Brain Connectivity Atlas	N/A
Gene Expression Data	Allen Mouse Brain Atlas	N/A
Perturbation Data	(Le Merre et al.)^24^	https://carlenlab.org/data/
Experimental models: Organisms/strains		
Mouse: C57BL/6J	Animal Care Committee at “The Center for Phenogenomics (TCP)”; Animal Use Protocol 26-0260H	N/A
Software and algorithms		
MATLAB 2021a	MathWorks	N/A
FMRIB Software Library (FSL) v6.0	Wellcome Center for Integrative Neuroimaging, FMRIB, University of Oxford	N/A
Deposited Software	This paper	https://doi.org/10.5281/zenodo.10912448
MrCat Toolbox	Cognitive Neuroecology Lab at the Radboud University Nijmegen and the University of Oxford	https://github.com/neuroecology/MrCat

### Resource Availability

#### Lead contact

Further information and requests for resources should be directed to and will be fulfilled by the Lead Contact, Alberto Lazari (alberto. lazari@ndcn.ox.ac.uk).

#### Materials availability

This study did not generate new unique reagents.

### Experimental Model and Study Participant Details

#### Animal models

20 wildtype C57BL/6J mice male adult mice (age ranging from P80 to P90) were used to collect Multi-Parameter Mapping (MPM) data. All experiments conformed to the relevant regulatory standards (Animal Care Committee at “The Center for Phenogenomics (TCP)”; Animal Use Protocol 26-0260H).

### Method Details

#### Resting-state functional MRI (rs-fMRI) data acquisition

##### Mouse

We used existing data from 20 wildtype C57BL/6J mice (all male, average age: P112) that underwent a single rs-fMRI scanning session, as described in the original studies.^46,119^ Animals were caged in standard housing (maximum 5 animals/cage), with food and water *ad libitum*, and a 12 h day/night cycle. All MRI scans were conducted in the light phase. All experimental protocols were carried out under licensing from the Zuürich Cantonal veterinary office, and in accordance with the Swiss federal guidelines for the use of animals in research.

Mouse rs-fMRI was collected using a BioSpec 70/16 (7T field strength, 16 cm bore diameter) small animal MR system (Bruker BioSpin MRI, Ettlingen, Germany) with a cryogenic quadrature surface coil (Bruker BioSpin AG, Fällanden, Switzerland). A gradient-echo EPI sequence (GE-EPI, repetition time TR = 1000 ms, echo time TE = 15 ms, in-plane resolution = 0.22 × 0.2 mm2, number of slice = 20, slice thickness = 0.4 mm, slice gap = 0.1 mm) was applied to acquire 900 volumes. rs-fMRI scanning was conducted under anesthesia, with levels of anesthesia and mouse physiological parameters being monitored following an established protocol to obtain a reliable measurement of functional connectivity.^40,119^

##### Human

20 volumetric resting state fMRI datasets (10 male; 5 in the 22–25 age range; 10 in the 26–30 age range; 5 in the 31–35 age range) were downloaded from the Human Connectome Project (HCP).^45^ In brief, the HCP rs-fMRI acquisition consisted of whole-brain BOLD EPI images collected (1200 volumes) using a standardised protocol (2 mm isotropic resolution, 72 slices, TR = 720 ms, TE = 33.1 ms, multiband factor = 8). For each subject, only the first session with phase encoding left-to-right (LR) was used.

#### Multi-parameter mapping (MPM) data acquisition and preprocessing

##### Mouse

20 wildtype C57BL/6J mice male adult mice (age ranging from P80 to P90) were sacrificed and perfused with 4% PFA, without additional contrast agents, and following a perfusion protocol optimised for *ex vivo* MR imaging.^120,121^ Prior to perfusion, mice were housed in standard group housing, minimally handled, and received food and water *ad libitum*, in order to reduce environmental influences on myelination. Before scanning, brains were checked for the presence of air bubbles that may distort the images; 5 mouse brains contained bubbles and were thus not scanned. An additional 3 mouse brains were damaged through accidental freezing and were thus excluded from the study.

Mouse Multi-Parametric Mapping (MPM) data was collected on a 7T BioSpec 70/20 USR small animal MR system with a Paravision 360 console (Bruker BioSpin MRI, Ettlingen, Germany) using an 86mm transmit volume coil (Bruker BioSpin MRI, Germany) in combination with a receive-only cryoprobe 2 × 2 actively detuned elements array (Bruker BioSpin AG, Switzerland). Bore temperature was set at 22°C.

The mouse *ex vivo* MPM protocol was adapted from a human *in vivo* protocol.^52^ It included 18 repetitions of a set of three multiecho 3D FLASH (Fast Low-Angle Shot) scans as well as two DAM (Double Angle Mapping) scans for B1 mapping, for a total acquisition time of roughly 8 h. Each set of 3D FLASH scans consisted of T1-, PD- (Proton Density), and MT- (Magnetisation Transfer) weighted imaging. The FA (Flip Angle) was set to 30 deg for T1 and to 6 for PD and MT scans. For each scan, 100 μm isotropic images were acquired using a Field of View (FOV) of 24 × 6.9 × 12 mm^3^, a bandwidth of 100 kHz, and TR/TE1 = 51/3 ms. Eight echos or six echos with an inter-echo delay of 4 ms were acquired for T1/PD and MT, respectively. The MT preparation consisted of an off-resonance Gauss RF pulse with a magnitude of 10microT and a frequency offset of 2 kHz. To correct for field drift during the scanning, a navigator signal acquired for each TR with a non-selective Gauss RF pulse of 1 deg was used to prospectively update the resonance frequency. The acquisition parameters were as follows: FOV = 19 × 10 mm^2^, spatial resolution = 250 × 250 μm,^10^ 46 slices with a thickness of 250 μm, bandwidth = 100 kHz, and TR/TE = 7500/3 ms.

To correct for B1 inhomogeneities, DAM (Double Angle Mapping) technique with FAs of 40 and 80 deg was used to map B1+ field.^122^ The equation to derive the B1+ map was as follows:

(Equation 1) $$\text{B}1+=\frac{180}{\pi *A1\;\text{angle}\;}\times \arccos\frac{A2}{A1*2}\times 100$$

where B1+ is the B1+ value expressed as a percentage, A1 is the lower flip angle scan, A2 is the higher flip angle scan, and A1 _angle_ is the flip angle of A1 (in this case, 40°).

To correct for hardware drift during the acquisition, a multi-step co-registration procedure was performed. First, echoes were averaged within each repetition and for each contrast. Second, these average images were coregistered to the average of the corresponding contrast of the first repetition with a 6 degrees of freedom Normalised Mutual Informatiion cost function in FSL’s FLIRT.^123^ Third, the resulting transformation matrices were applied to all individual echoes of all repetitions. Fourth, once all echoes were thus transformed into a common space, they were averaged across repetitions, to create a high-SNR image for each echo of the acquisition protocol.

Separately, the B1+ map was calculated using the DAM method with fslmaths tools^123^ and co-registered to the average of the first repetition using the same registration approach described above. The high-SNR average echoes and B1+ map were then used to estimate Magnetisation Transfer saturation (MT), R1 and R2* quantitative maps through the hMRI toolbox.^124^

##### Human

We used existing MPM scans from 50 participants collected using a 3T Prisma Magnetom Siemens scanner, software version VE11C (Siemens Medical Systems, Erlangen, Germany), as described in the original study.^9^ The MPM protocol was based on^52^ and included three multi-echo 3D FLASH (fast low-angle shot) scans with varying acquisition parameters, one RF transmit field map (B1+map) and one static magnetic (B0) field map scan, for a total acquisition time of roughly 22 min. To correct for inter-scan motion, position-specific receive coil sensitivity field maps, matched in FOV to the MPM scans, were calculated and corrected for.^125^

The three 3D FLASH scans were designed to be predominantly T1-, PD-, or MT-weighted by changing the flip angle and the presence of a pre-pulse: 8 echoes were predominantly Proton Density-weighted (TR = 25 ms; flip angle = 6°; TE = 2.3–18.4 ms), 8 echoes were predominantly T1-weighted (TR = 25 ms; flip angle = 21°; TE = 2.3–18.4 ms) and 6 echoes were predominantly Magnetisation Transfer-weighted (MTw, TR = 25ms; flip angle = 21°; TE = 2.3–13.8 ms). For MTw scans, excitation was preceded by off-resonance Gaussian MT pulse of 4 ms duration, nominal flip angle, 2 kHz frequency offset from water resonance. All FLASH scans had 1 mm isotropic resolution, field of view (FOV) of 256 × 224 × 176 mm^3^, and GRAPPA factor of 2 × 2. The B1 map was acquired through an EPI-based sequence featuring spin and stimulated echoes (SE and STE) with 11 nominal flip angles, FOV of 192 × 192 × 256 mm^3^ and TR of 500 ms. The TE was 37.06 ms, and the mixing time was 33.8 ms. The B0 map was acquired to correct the B1+ map for distortions due to off-resonance effects. The B0 map sequence had a TR of 1020.0 ms, first TE of 10 ms, second TE of 12.46 ms, field of view (FOV) of 192 × 192 × 256 mm^3^ and readout bandwidth of 260 Hz/pixel. Magnetisation Transfer saturation (MT), R1 and R2* quantitative maps were estimated through the hMRI toolbox^124^ and then registered to Montreal Neurological Institute (MNI) space.

##### Tracer data

Normalised Fluorescence maps from Allen Mouse Brain Connectivity Atlas^28^ were downloaded and preprocessed.^126^ The Allen Mouse Brain Connectivity Atlas uses Adeno-Associated Viral vectors (AAVs) to systematically track long-range connections in the mouse brain,^28^ and is thus suitable to answer questions about whole-brain connectivity patterns in the mouse motor network. In brief, 3D maps from tracer injection experiments in wild-type mice were downloaded, resampled to 0.2 isotropic resolution and normalised to a 0–1 intensity distribution. After registration to the Queensland Brain Institute (QBI) atlas, the tracing maps were concatenated and used as input for the k-means clustering algorithm.

##### Gene expression data

Mouse gene expression data was extracted from the adult mouse whole-brain *in situ* hybridization datasets of the Allen Mouse Brain Atlas.^47^ The coronal *in situ* hybridization images from 3958 mouse genes were preprocessed and normalized,^109^ without excluding any further gene *a priori*. We then computed the average expression of each gene across the voxels in each of the premotor and motor areas.

##### Perturbation data and behavior

An openly available database of 100 previously-published inactivation experiments^24^ was used to study inactivation of aM2 and pM2 (https://carlenlab.org/data/). The database contains information from experiments targeting mouse frontal areas and establishing impairments in a wide range of task-based behaviors. To analyze the tasks in an unbiased way, we used pre-specified descriptors from the database itself, and assessed task features with the following categories: sensorimotor transformation (task type set as “sensorimotor transformation” in the original database), context-based task (task type set as “context/rule-based task” in the original database), memory (task type set as “memory/delay” in the original database), task complexity (complexity index above 5 in the original database; a full description of the rationale and scoring for the complexity index are provided in the original publication and related database^24^), movement quantity (number of actions above 1 in the original database), and complexity of actions (movement complexity equal to 1 in the original database).

##### Pre-processing of rs-fMRI data

rs-fMRI data was preprocessed with a common ICA-denoising approach across mouse and human data.^40,41,127^ After registration to standard space, scans were smoothed by twice the voxel size in the XY dimension (resulting in a sigma of 0.4 mm for mouse data and of 4 mm for human data).

##### Defining motor and premotor seeds in mouse and human

In humans, motor and premotor seeds were identified using the Glasser atlas^128^ for M1, FEF, PMd, PMv. These were complemented with more fine-grained comparative parcellations for pre-SMA and SMA,^60^ and for RCZa and RCZp which form Medial Cingulate Cortex.^31^ In mice, motor and premotor areas were defined through an axonal tracer-driven parcellation of voxels from traditional M1 and M2, as defined in the DSURQE atlas,^51^ as described in the Results. Relevant code is available here: https://git.fmrib.ox.ac.uk/preclinical-imaging/premotor-mouse-human.

##### Defining connectivity targets in mouse and human

A set of connectivity targets was chosen *a priori* by inspecting 165 mouse brain regions from the Allen Brain Atlas ontology and selecting those that are considered to have high homology to human areas. The resulting list largely overlapped with previous literature,^41^ as follows: 1) Primary Somatosensory Cortex (S1), 2) Supplemental Somatosensory Cortex (S2), 3) Primary Visual Cortex (V1), 4) Primary Auditory Cortex (A1), 5) Dorsal (in the human, Anterior) Hippocampus, 6) Ventral (in the human, Posterior) Hippocampus, 7) Infralimbic (IL; in the human, Area 25), 8) Prelimbic (PL; in the human, Area 32), 9) Retrosplenial Cortex (RSC; in the human, Area 30), 10) Lateral Orbitofrontal Cortex (Area 13), 11) Basolateral Amgydala, 12) Temporal association area (TPJp). Based on recently discovered striatal homologies between mice and humans,^41^ 13) medial Caudoputamen (CPm) 14) lateral Caudoputamen (CPl) and 15) Nucleus Accumbens (NAcc) were added to the list of targets. Finally, the majority of target regions of interest were created in the right hemisphere. However, as premotor areas are known to have asymmetric connectivity across hemispheres in primates, we decided to also include a selected number of left hemisphere ROIs, namely 16) left primary somatosensory cortex (S1), 17) left secondary somatosensory cortex (S2) and 18) left primary motor cortex (M1, as defined in previous studies and corresponding to M1), with the goal of better distinguishing between premotor areas in both species. Targets were created as 3 × 3 × 3 voxels in all species; further information on the precise coordinates that were used is in the supplemental information. ROI files are available here: https://git.fmrib.ox.ac.uk/preclinical-imaging/premotor-mouse-human.

### Quantification and Statistical Analysis

All of the statistical details of experiments can be found in the Results, Figures, Figure Legends and supplemental information.

#### Fingerprint-based comparative analyses

For each mouse seed, connectivity fingerprints were extracted from individual scans and the median of all fingerprints was derived to create a ‘template connectivity fingerprint’ for each seed. Then, for each voxel of each human scan, we extracted a connectivity fingerprint, correlated the human connectivity fingerprint to the mouse template connectivity fingerprint, and finally assigned the correlation effect size to the voxel in question. This process resulted in multiple voxel-wise maps (one per mouse seed) for each human scan. All maps were Fisher’s r-to-Z transformed. Maps related to a single mouse seed were then run together through permutation testing in FSL’s randomise (1,000 permutations, TFCE family-wise error corrected *p* < 0.05) to establish which voxels showed a significantly similar connectivity fingerprint.^41^

#### Winner-take-all and spectral clustering analyses

Winner-take-all analyses were run by comparing the four fingerprint matches of each human voxel (M1, ALM, aM2, pM2), and assigning that voxel to the mouse area with the strongest fingerprint match, as measured by the t-statistic of a fingerprint-based permutation test (as described above). Winner-take-all analyses were across a custom motor network mask designed to include as many putative motor network voxels as possible, including the full precentral gyrus, as well as clustering-based parcellations of pre-SMA and SMA,^60^ and of Mid Cingulate Cortex (RCZa and RCZp).^31^

Spectral clustering analyses were run for mouse-human comparisons.^30^ In short, connectivity fingerprints of each mouse and human motor and premotor areas were used to calculate a Kullback-Leibler (KL)-based divergence matrix. Spectral clustering^65^ was then used to map the divergence matrix in a 2D-space, thus clustering together regions with the similar connectivity fingerprints, independently of the species from which the connectivity fingerprint originated.

#### Additional statistical analyses

In order to confirm the difference between connectivity, cortical myelin and gene expression profiles of different premotor subdivisions in the mouse, the MrCat toolbox (https://github.com/neuroecology/MrCat) was used to establish the Manhattan distance and perform permutation testing (10,000 permutations) to test for significance.^59^ In addition, paired sample t-tests were used to compare myelin markers between two regions of interest.

## Supplementary Material

Supplemental information can be found online at https://doi.org/10.1016/j.celrep.2024.114191.

Supplemental information

## Data Availability

Raw data reported in this paper will be shared by the lead contact upon request. All original code has been deposited here: (https://git.fmrib.ox.ac.uk/preclinical-imaging/premotor-mouse-human/, https://doi.org/10.5281/zenodo.10912448), and is publicly available as of the date of publication. Any additional information required to reanalyze the data reported in this paper is available from the lead contact upon request.

## Highlights

- Combined multimodal data reveal multiple premotor areas within the mouse motor network
- Three mouse premotor areas (ALM, aM2, pM2) have distinct structural and functional properties
- ALM has striking similarity to human ventral premotor areas, such as PMv
- aM2 and pM2 amalgamate properties of human pre-SMA and human cingulate cortex

## In brief

Lazari et al. provide evidence for the existence of multiple premotor areas within the mouse motor network. Three premotor areas, ALM, aM2, and pM2, have distinct structural, functional, and behavioral properties and show important similarities to and differences from the human motor system.
