# Supplementary material for: The mouse motor system contains multiple premotor areas and partially follows human organizational principles
Source: Cell Rep. Author manuscript; Available in PMC 2025 Jul 30. (PMC7617973; doi:10.1016/j.celrep.2024.114191)
Supplement: Supplemental information [file EMS207325-supplement-Supplemental_information.pdf]

Cell Reports, Volume *43*

Supplemental information

The mouse motor system contains multiple premotor areas and partially follows human organizational principles

Alberto Lazari, Mohamed Tachrount, Juan Miguel Valverde, Daniel Papp, Antoine Beauchamp, Paul McCarthy, Jacob Ellegood, Joanes Grandjean, Heidi Johansen-Berg, Valerio Zerbi, Jason P. Lerch, and Rogier B. Mars

Supplementary Information

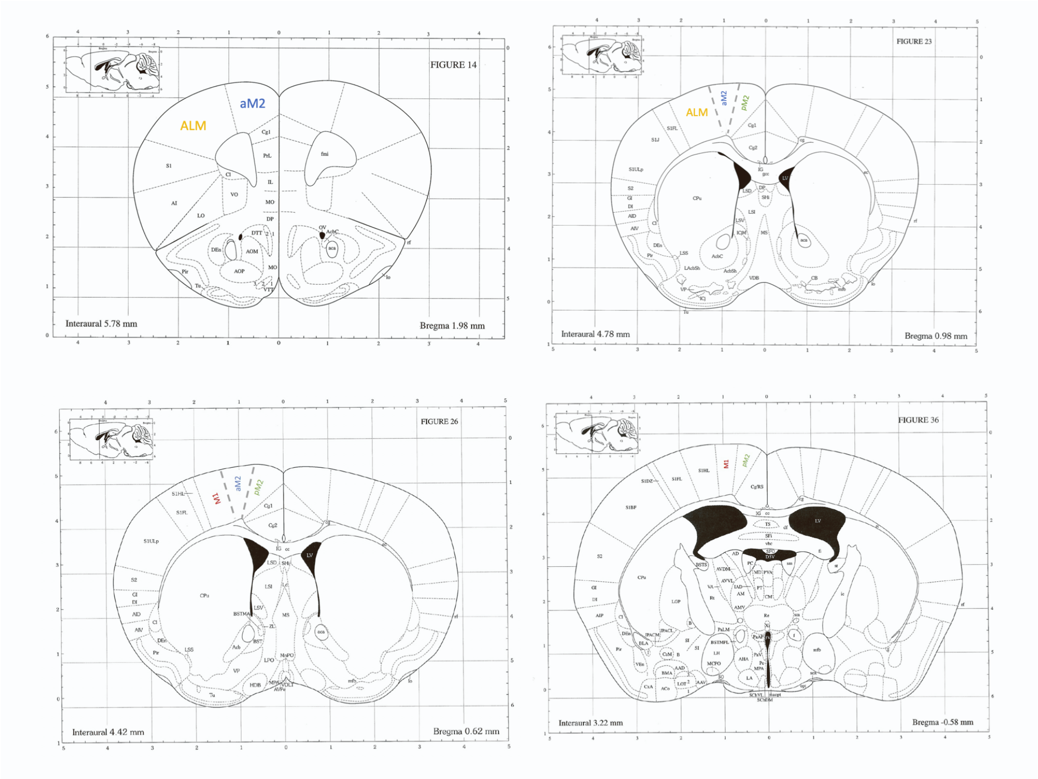

Supplemental Figure 1: **Updated Paxinos Atlas for stereotaxic surgeries on M1-RFA, ALM, aM2 and pM2.** Example figures from a 32-page Paxinos atlas resource created based on the axonal tracer parcellation, and available online (https://git.fmrib.ox.ac.uk/preclinical-imaging/premotor-mouse-human/). Related to Figure 2.

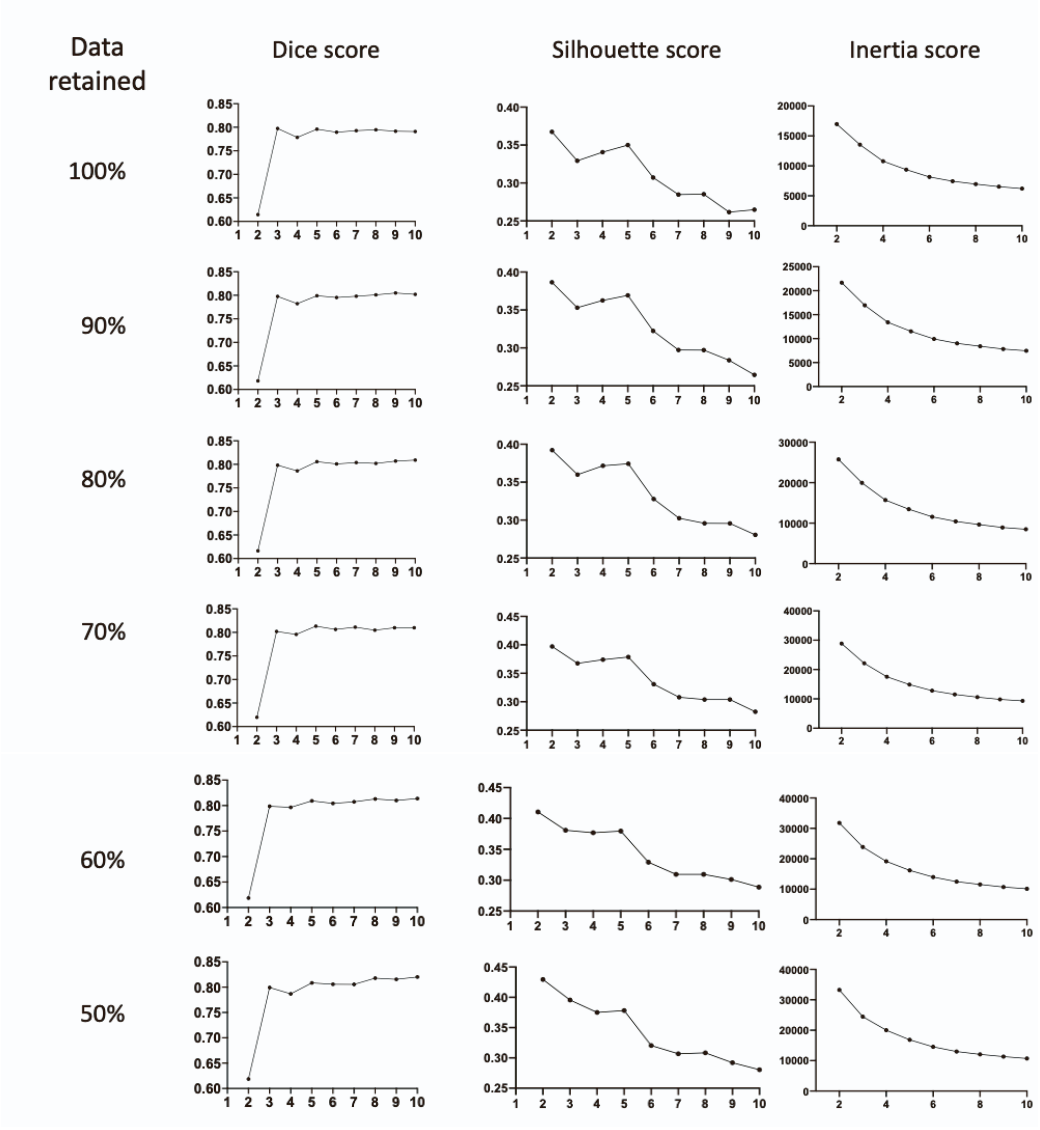

Supplemental Figure 2: **Assessment of tracer-based parcellations through different preprocessing** Different thresholds were applied to remove volumes from the overall tracer dataset (leftmost column). k-means clustering was then applied to the data, and for each k, the following metrics were calculated: Dice coefficient with the DSURQE M1-M2 division (centre-left column), Silhouette score (centre-right column), inertia (rightmost column). Overall, the results of our parcellations are robust to different data removal thresholds. Related to Figure 2.

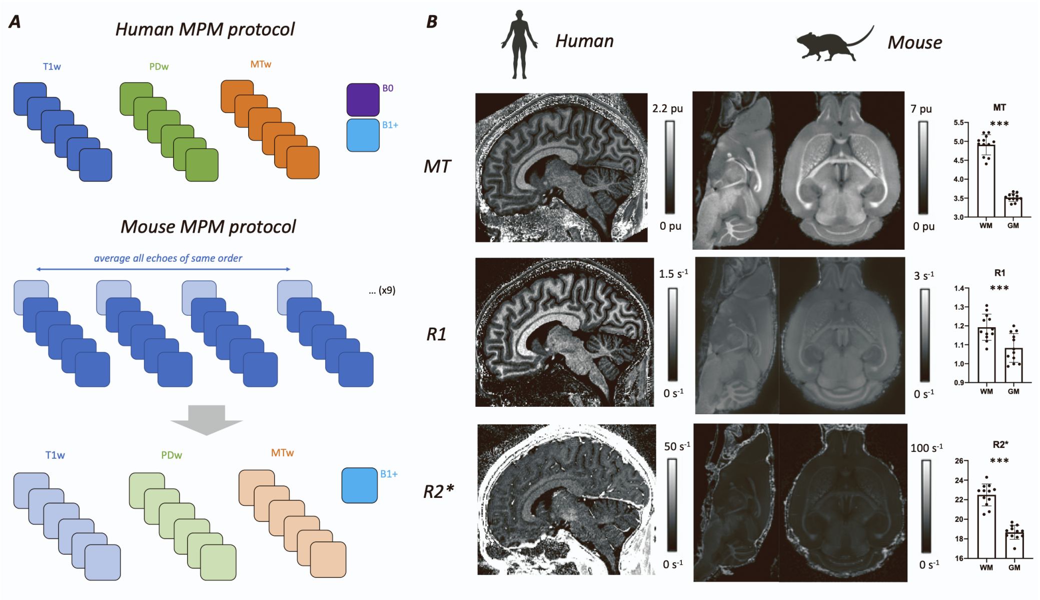

Supplemental Figure 3: **Multi-Parameter Mapping (MPM) for cross-species quantitative MRI.** A. A 3T human MPM sequence was adapted for use on a 7T preclinical scanner. Repetition of the sequence were averaged in order to achieve similar signal-to-noise ratio across species. B. Examples of single-subject quantitative MRI scans for the metrics used in the study (MT, R1, R2*). In mouse maps, values in Grey Matter (GM, region of interest in orbitofrontal cortex) where lower than in White Matter (WM, region of interest in corpus callosum; MT: $t=23.16$, $p<0.0001$; R1: $t=15.99$, $p<0.0001$; R2*: $t=12.97$, $p<0.0001$). $n = 12$, mean $\pm$ SD, paired sample t-test. Related to Figures 2 and 4.

Reference	Area Targetted	Sample Size	Task(s)
Huda et al., Nat Commun, 2020	pM2	29	Visual forepaw orienting task
Itokazu et al., Nat Commun, 2018	pM2	N/A	Sacade task
Leinweber et al., Neuron, 2017	pM2	7	2D Virtual navigation task and reversal of the optic folw (mismatch)
Li et al., PNAS, 2020	pM2	6	Delayed Go/noGo Auditory
Schneider et al., Nature, 2014	pM2	8	Running on a treadmill
Gilad et al., Neuron, 2018	pM2	3	Texture discrimination
Goard et al., Elife, 2016	pM2	4	Go/NoGo delayed visual task
Siniscalchi et al, Nat Neurosci, 2016	pM2	11	Adaptative decision making task (=rule switching)
White et al., Cell Rep, 2018	pM2	16	5-choice serial reaction time task
Manita et al., Neuron, 2015	aM2	8	Spontaneous Test Preference
Manita et al., Neuron, 2015	aM2	8	Tactile discrimination task
Xu et al., Nature, 2022	aM2	7	Sequence licking task
Allen et al., Neuron, 2017	aM2	10	Olfactory go/no-go decision-making task
Guo et al., Neuron, 2014	aM2	6	Whisker-based object localization task
Inagaki et al., J.Neurosci, 2018	aM2	5	Auditory delayed discrimination task
Inagaki et al., Nature, 2019	aM2	6	Auditory delayed discrimination task
Li et al., Nature, 2015	aM2	8	Whisker-based object localization task
Li et al., Nature, 2016	aM2	5	Whisker-based object localization task
Mayrhofer et al., Neuron, 2019	aM2	8	Perceptual decision making
Wu et al., Neuron, 2020	aM2	14	Olfactory delayed match to sample
Zimmermann et al., Biol. Psychiatry, 2017	aM2	N/A	Reinforcement learning
Makino et al., Neuron, 2017	aM2	7	Lever-press (auditory cued) motor task
Baltz et al., eLife, 2018	aM2	19	Incentive learning task
Morandell et al., Scientific reports, 2017	aM2	9	Directional joystick task

Supplementary Table 1. **Summary details of the 24 inactivation experiments analysed in Fig. 2I.** We report area targetted, sample size of each experiment, and a brief description of the task where the behavioural effect of stimulation was observed. Task description is reported as described by previous literature[54]. As the paper by Manita et al., 2015 contained two experiments, these were reported separately. Experiments targetting pM2 had a pooled sample size of 84 mice, whereas experiments targetting aM2 had a pooled sample size of 120 mice. Two included studies did not report sample size of animals used, and were thus excluded from the calculation of pooled sample sizes.

	Human Label	Mouse label	Franklin & Paxinos			Reference
			x	y	z	
CINGULATE	Area 25	IL	0.2	2.75	1.54	(Heilbronner et al., 2016; Vogt and Paxinos, 2014; Laubach et al., 2018)
	Area 32pl	PL	0.25	2	2.22	(Heilbronner et al., 2016; Vogt and Paxinos, 2014; Laubach et al., 2018)
	Area 24	CG1+2	0.15	2	0.98	(Heilbronner et al., 2016; Vogt and Paxinos, 2014; Laubach et al., 2018)
	Retrosplenial cortex	Retrosplenial area, ventral + dorsal part	1.25	1	-1.94	(Vogt and Paxinos, 2014)
ORBITALFRONTAL	Area 13	Orbitofrontal lateral	1.25	2.5	2.46	(Wise, 2008)
AMYG	Basolateral Amygdala	Basolateral amygdalar nucleus	2.9	4.7	-2.06	(Heilbronner et al., 2016)
HIPPO	Anterior Hippocampus	Ventral Hippocampus	2.25	2.5	-3.6	(Strange et al., 2014)
	Posterior Hippocampus	Dorsal Hippocampus	0.5	2.1	-1.58	(Strange et al., 2014)
SOMATOSENSORY	S1	average of somatosensory areas	2.5	1.5	0.26	(Balleine and O'Doherty, 2010; Liska et al., 2015)
	S2 (OP1)	Supplemental somatosensory area	3.5	3	-0.22	(Balleine and O'Doherty, 2010; Liska et al., 2015)
TEMPORAL	TPJp	Temporal Association Area	4.1	3.1	-2.8	(Grandjean et al., 2019; Zerbi et al., 2015)
STRIATUM	posterior putamen	Lateral caudoputamen (CPl)	2.5	3.75	0.02	(Balsters et al., 2020; McCutcheon et al., 2019)
	caudate nucleus	Medial caudoputamen (CPm)	1	3	0.98	(Balsters et al., 2020; McCutcheon et al., 2019)
	nucleus accumbens	Nucleus Accumbens (Nacc)	0.75	4.75	0.98	(Balsters et al., 2020; McCutcheon et al., 2019)
AUDITORY	A1	Primary auditory cortex	3.75	2.25	-2.92	(Krubitzer and Seelke, 2012; Guo et al., 2012)
VISUAL	V1	Primary visual cortex	2.5	1	-3.64	(Krubitzer and Seelke, 2012; Gordon and Stryker, 1996)

Supplementary Table 2. **Summary of mouse and human target areas.** Franklin and Paxinos coordinates are reported for mouse seeds (Franklin and Paxinos, 2019). References are provided to support homologies across species.

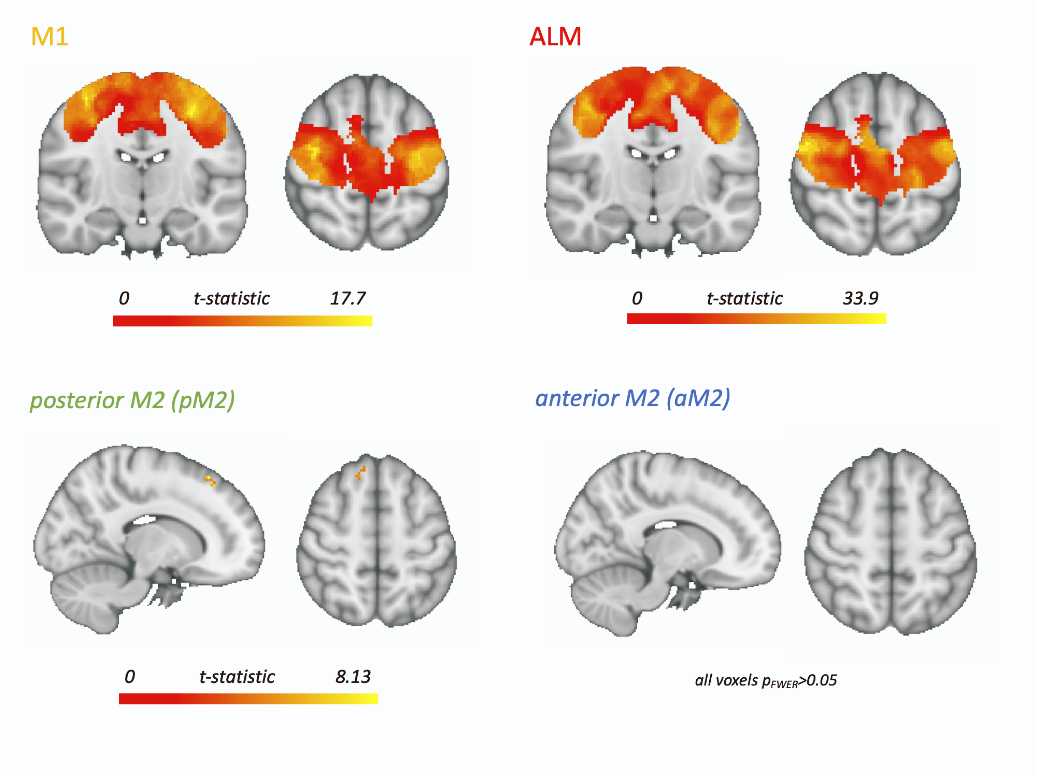

Supplemental Figure 4: **Voxelwise fingerprint-matching results for each mouse premotor subdivision** T-statistic maps obtained from permutation tests were thresholded at $p_{FWER} < 0.05$. Results from this analysis were fed into a winner-takes-all algorithm that assigned each voxel to the subdivision with the highest t-statistic score. Related to Figure 4.

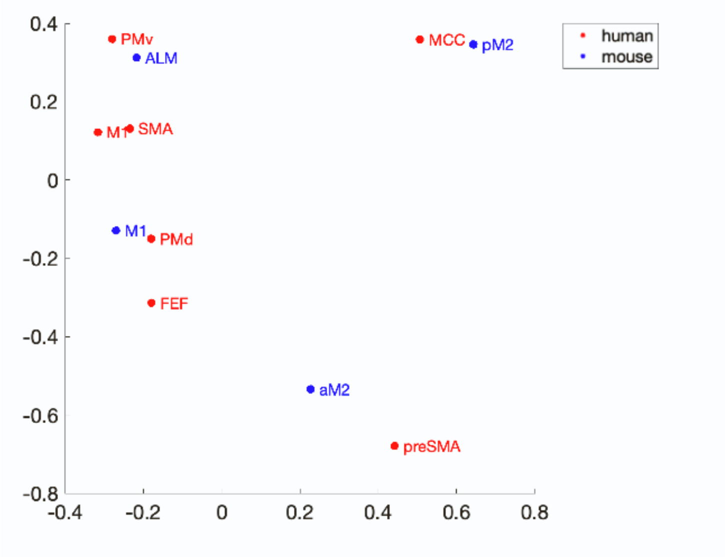

Supplemental Figure 5: **Spectral clustering without including information from primary visual and auditory cortex. Related to Figure 4.**

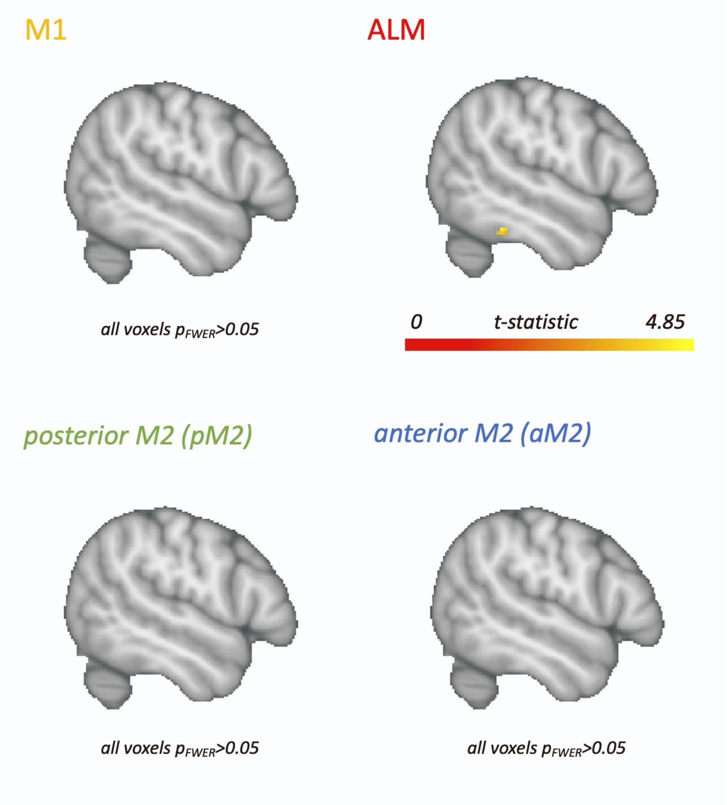

Supplemental Figure 6: **Control analyses for voxelwise fingerprint-matching results.** The fingerprint-matching analysis was run for all mouse motor network subdivisions within a human control region of interest (inferior temporal gyrus). T-statistic maps obtained from permutation tests were thresholded at $p_{FWER} < 0.05$. Related to Figure 4.

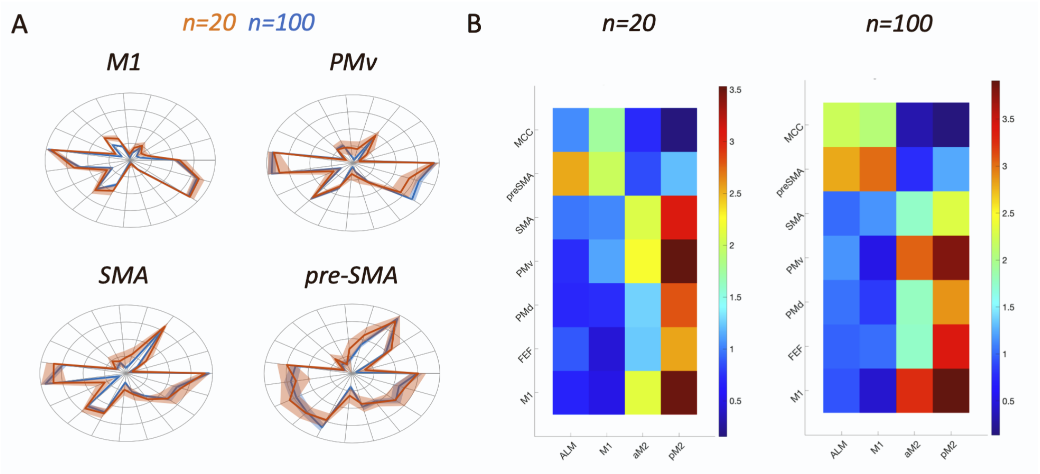

Supplemental Figure 7: textbfComparison of results at different sample sizes A. Connectivity fingerprints are similar at n=20 and n=100. B. KL divergence patterns are similar at n=20 and n=100. Related to Figure 4.

Reference List for Supplementary Table 1

Balleine, B. W. and O'doherty, J. P. (2010). Human and rodent homologies in action control: corticostriatal determinants of goal-directed and habitual action. *Neuropsychopharmacology*, 35(1):48–69.

Balsters, J. H., Zerbi, V., Sallet, J., Wenderoth, N., and Mars, R. B. (2020). Primate homologs of mouse cortico-striatal circuits. *Elife*, 9:e53680.

Gordon, J. A. and Stryker, M. P. (1996). Experience-dependent plasticity of binocular responses in the primary visual cortex of the mouse. *Journal of Neuroscience*, 16(10):3274–3286.

Grandjean, J., Canella, C., Anckaerts, C., Ayrancı, G., Bougacha, S., Bienert, T., Buehlmann, D., Coletta, L., Gallino, D., Gass, N., et al. (2020). Common functional networks in the mouse brain revealed by multi-centre resting-state fmri analysis. *Neuroimage*, 205:116278.

Guo, W., Chambers, A. R., Darrow, K. N., Hancock, K. E., Shinn-Cunningham, B. G., and Polley, D. B. (2012). Robustness of cortical topography across fields, laminae, anesthetic states, and neurophysiological signal types. *Journal of Neuroscience*, 32(27):9159–9172.

Heilbronner, S. R., Rodriguez-Romaguera, J., Quirk, G. J., Groenewegen, H. J., and Haber, S. N. (2016). Circuit-based corticostriatal homologies between rat and primate. *Biological psychiatry*, 80(7):509–521.

Krubitzer, L. A. and Seelke, A. M. (2012). Cortical evolution in mammals: the bane and beauty of phenotypic variability. *Proceedings of the National Academy of Sciences*, 109(supplement_1):10647–10654.

Laubach, M., Amarante, L. M., Swanson, K., and White, S. R. (2018). What, if anything, is rodent prefrontal cortex? *eneuro*, 5(5).

Liska, A., Galbusera, A., Schwarz, A. J., and Gozzi, A. (2015). Functional connectivity hubs of the mouse brain. *Neuroimage*, 115:281–291.

McCutcheon, R. A., Abi-Dargham, A., and Howes, O. D. (2019). Schizophrenia, dopamine and the striatum: from biology to symptoms. *Trends in neurosciences*, 42(3):205–220.

Strange, B. A., Witter, M. P., Lein, E. S., and Moser, E. I. (2014). Functional organization of the hippocampal longitudinal axis. *Nature reviews neuroscience*, 15(10):655–669.

Vogt, B. A. and Paxinos, G. (2014). Cytoarchitecture of mouse and rat cingulate cortex with human homologies. *Brain Structure and Function*, 219:185–192.

Wise, S. P. (2008). Forward frontal fields: phylogeny and fundamental function. *Trends in neurosciences*, 31(12):599–608.

Zerbi, V., Grandjean, J., Rudin, M., and Wenderoth, N. (2015). Mapping the mouse brain with rs-fmri: An optimized pipeline for functional network identification. *Neuroimage*, 123:11–21.

Reference List for Supplementary Table 2

Allen, W. E., Kauvar, I. V., Chen, M. Z., Richman, E. B., Yang, S. J., Chan, K., Gradinaru, V., Deverman, B. E., Luo, L., and Deisseroth, K. (2017). Global representations of goal-directed behavior in distinct cell types of mouse neocortex. *Neuron*, 94(4):891–907.

Baltz, E. T., Yalcinbas, E. A., Renteria, R., and Gremel, C. M. (2018). Orbital frontal cortex updates state-induced value change for decision-making. *Elife*, 7:e35988.

Gilad, A., Gallero-Salas, Y., Groos, D., and Helmchen, F. (2018). Behavioral strategy determines frontal or posterior location of short-term memory in neocortex. *Neuron*, 99(4):814–828.

Goard, M. J., Pho, G. N., Woodson, J., and Sur, M. (2016). Distinct roles of visual, parietal, and frontal motor cortices in memory-guided sensorimotor decisions. *elife*, 5:e13764.

Guo, Z. V., Li, N., Huber, D., Ophir, E., Gutnisky, D., Ting, J. T., Feng, G., and Svoboda, K. (2014). Flow of cortical activity underlying a tactile decision in mice. *Neuron*, 81(1):179–194.

Huda, R., Sipe, G. O., Breton-Provencher, V., Cruz, K. G., Pho, G. N., Adam, E., Gunter, L. M., Sullins, A., Wickersham, I. R., and Sur, M. (2020). Distinct prefrontal top-down circuits differentially modulate sensorimotor behavior. *Nature communications*, 11(1):6007.

Inagaki, H. K., Fontolan, L., Romani, S., and Svoboda, K. (2019). Discrete attractor dynamics underlies persistent activity in the frontal cortex. *Nature*, 566(7743):212–217.

Inagaki, H. K., Inagaki, M., Romani, S., and Svoboda, K. (2018). Low-dimensional and monotonic preparatory activity in mouse anterior lateral motor cortex. *Journal of Neuroscience*, 38(17):4163–4185.

Itokazu, T., Hasegawa, M., Kimura, R., Osaki, H., Albrecht, U.-R., Sohya, K., Chakrabarti, S., Itoh, H., Ito, T., Sato, T. K., et al. (2018). Streamlined sensory motor communication through cortical reciprocal connectivity in a visually guided eye movement task. *Nature communications*, 9(1):1–14.

Leinweber, M., Ward, D. R., Sobczak, J. M., Attinger, A., and Keller, G. B. (2017). A sensorimotor circuit in mouse cortex for visual flow predictions. *Neuron*, 95(6):1420–1432.

Li, B., Nguyen, T. P., Ma, C., and Dan, Y. (2020). Inhibition of impulsive action by projection-defined prefrontal pyramidal neurons. *Proceedings of the National Academy of Sciences*, 117(29):17278–17287.

Li, N., Chen, T.-W., Guo, Z. V., Gerfen, C. R., and Svoboda, K. (2015). A motor cortex circuit for motor planning and movement. *Nature*, 519(7541):51–56.

Li, N., Daie, K., Svoboda, K., and Druckmann, S. (2016). Robust neuronal dynamics in premotor cortex during motor planning. *Nature*, 532(7600):459–464.

Makino, H., Ren, C., Liu, H., Kim, A. N., Kondapaneni, N., Liu, X., Kuzum, D., and Komiyama, T. (2017). Transformation of cortex-wide emergent properties during motor learning. *Neuron*, 94(4):880–890.

Manita, S., Suzuki, T., Homma, C., Matsumoto, T., Odagawa, M., Yamada, K., Ota, K., Matsubara, C., Inutsuka, A., Sato, M., et al. (2015). A top-down cortical circuit for accurate sensory perception. *Neuron*, 86(5):1304–1316.

Mayrhofer, J. M., El-Boustani, S., Foustoukos, G., Auffret, M., Tamura, K., and Petersen, C. C. (2019). Distinct contributions of whisker sensory cortex and tongue-jaw motor cortex in a goal-directed sensorimotor transformation. *Neuron*, 103(6):1034–1043.

Morandell, K. and Huber, D. (2017). The role of forelimb motor cortex areas in goal directed action in mice. *Scientific reports*, 7(1):15759.

Schneider, D. M., Nelson, A., and Mooney, R. (2014). A synaptic and circuit basis for corollary discharge in the auditory cortex. *Nature*, 513(7517):189–194.

Siniscalchi, M. J., Phoumthipphavong, V., Ali, F., Lozano, M., and Kwan, A. C. (2016). Fast and slow transitions in frontal ensemble activity during flexible sensorimotor behavior. *Nature Neuroscience*, 19(9):1234–1242.

White, M. G., Panicker, M., Mu, C., Carter, A. M., Roberts, B. M., Dharmasri, P. A., and Mathur, B. N. (2018). Anterior cingulate cortex input to the claustrum is required for top-down action control. *Cell reports*, 22(1):84–95.

Wu, Z., Litwin-Kumar, A., Shamash, P., Taylor, A., Axel, R., and Shadlen, M. N. (2020). Context-dependent decision making in a premotor circuit. *Neuron*, 106(2):316–328.

Xu, D., Dong, M., Chen, Y., Delgado, A. M., Hughes, N. C., Zhang, L., and O'Connor, D. H. (2022). Cortical processing of flexible and context-dependent sensorimotor sequences. *Nature*, 603(7901):464–469.

Zimmermann, K. S., Yamin, J. A., Rainnie, D. G., Ressler, K. J., and Gourley, S. L. (2017). Connections of the mouse orbitofrontal cortex and regulation of goal-directed action selection by brain-derived neurotrophic factor. *Biological psychiatry*, 81(4):366–377.
